# New generalizations of Popoviciu-type inequalities via new Green’s functions and Montgomery identity

**DOI:** 10.1186/s13660-017-1379-y

**Published:** 2017-05-10

**Authors:** Nasir Mehmood, Ravi P Agarwal, Saad Ihsan Butt, Josip Pečarić

**Affiliations:** 1 Department of Mathematics, COMSATS, Institute of Information Technology, Lahore, Pakistan; 2 Department of Mathematics, Texas A&M University - Kingsville, 700 University Blvd., Kingsville, USA; 3 Faculty of Textile Technology, University of Zagreb, Zagreb, 10000 Croatia

**Keywords:** 26A51, 26D07, 26D15, 26D20, 26D99, Popoviciu’s inequality, Montgomery identity, Abel-Gontscharoff interpolating polynomial, new Green’s functions, Grüss upper bounds, Ostrowski-type bounds

## Abstract

The inequality of Popoviciu, which was improved by Vasić and Stanković (Math. Balk. 6:281-288, 1976), is generalized by using new identities involving new Green’s functions. New generalizations of an improved Popoviciu inequality are obtained by using generalized Montgomery identity along with new Green’s functions. As an application, we formulate the monotonicity of linear functionals constructed from the generalized identities, utilizing the recent theory of inequalities for n-convex functions at a point. New upper bounds of Grüss and Ostrowski type are also computed.

## Introduction

Higher order convexity was introduced by Popoviciu, who defined it under the context of divided differences of a function (see Ch.1, [2]). Inequalities of higher order convex functions are very important and many physicists used them while dealing with higher dimensions. It is interesting to note that results for convex functions may not be true for convex functions of higher order. There are remarkable changes in the results, which force to think about the existence of such results. Butt and Pečarić paid tribute to Professor T. Popoviciu in their book [3] in 2015 to commemorate 50 years to Popoviciu’s inequality. They generalized Popoviciu’s inequality for higher order convex functions and gave its applications. Also in 2015 [4], a new class of n-convex functions at a point was introduced by Pečarić, Praljak and Witkowski. They developed a remarkable theory to investigate linear operator inequalities with the help of the functions, which are n-convex at a point. This theory leads to many interesting and fascinating results with a lot of applications in operator theory and statistics. A characterization of convex functions established by Popoviciu [5] has been studied by many people (see [1, 2] and the references therein). In recent years the inequality of Popoviciu was studied in [6–9]. The following form of Popoviciu’s inequality is by Vasić and Stanković in [1] (see page 173 [2]).

### Theorem 1

*Let* $[\delta_{1},\delta_{2}]$ *be an interval in* $\mathbb {R}$, *for integers* $m\geq3$, $2\leq s\leq m-1$, *consider the* *m*-*tuples* $\mathbf{z}=(z_{1},\ldots,z_{m}) \in[\delta_{1},\delta_{2}]^{m}$, $\mathbf{q}=(q_{1},\ldots,q_{m})$ *be positive* *m*-*tuples along with the condition that* $\sum_{i = 1}^{m} {{q_{i}}} = 1$. *Then*, *for* $\psi:[\delta_{1},\delta_{2}]\rightarrow\mathbb{R}$ *being a convex function*,

1 $$ {\psi_{s,m}}(\mathbf{z},\mathbf{q}) \le\frac{{m - s}}{{m - 1}} { \psi _{1,m}}(\mathbf{z},\mathbf{q}) + \frac{{s - 1}}{{m - 1}}{\psi _{m,m}}(\mathbf{z},\mathbf{q}) $$

*holds*, *where*

$$ {\psi_{s,m}}(\mathbf{z},\mathbf{q}): = \frac{1}{{C_{s - 1}^{m - 1}}}\sum _{1 \le{i_{1}} < \cdots < {i_{s}} \le m} { \Biggl( {\sum_{j = 1}^{s} {{q_{{i_{j}}}}} } \Biggr)\psi \biggl( {\frac{{\sum_{j = 1}^{s} {{q_{{i_{j}}}}{z_{{i_{j}}}}} }}{{\sum_{j = 1}^{s} {{q_{{i_{j}}}}}} }} \biggr)} $$

*is the linear functional with respect to* *ψ*.

By inequality (1), we write

2 $$ \operatorname{POP}[\mathbf{z},\mathbf{q};\psi]:= \frac{{m - s}}{{m - 1}} {\psi_{1,m}}(\mathbf{z},\mathbf{q}) + \frac{{s - 1}}{{m - 1}}{\psi _{m,m}}(\mathbf{z},\mathbf{q}) - {\psi_{s,m}}(\mathbf{z}, \mathbf{q}). $$

### Remark 1

Under the assumptions of Theorem 1, $\operatorname {POP}[\mathbf{z},\mathbf{q};\psi]\ge0$ for *ψ* being a convex function and zero for a constant and linear function.

In order to obtain our main results in the present paper, we use the generalized Montgomery identity via Taylor’s formula given in paper [10].

### Theorem 2

*Let* $n\in\mathbb{N}$, $\psi:I\rightarrow\mathbb{R}$ *be such that* $\psi^{ ( n-1 )} $ *is absolutely continuous*, $I\subset \mathbb{R}$ *is an open interval*, $\delta_{1},\delta_{2}\in I$, $\delta_{1}<\delta_{2}$. *Then the following identity holds*:

3 $$\begin{aligned} \psi ( z ) & =\frac{1}{\delta_{2}-\delta_{1}} \int_{\delta _{1}}^{\delta_{2}}\psi ( \xi ) \,d\xi+\sum _{l=0}^{n-2}\frac{\psi^{ ( l+1 )} (\delta_{1} )}{l! (l+2 )} \frac{ ( z-\delta_{1} ) ^{l+2}}{\delta _{2}-\delta_{1}}-\sum _{l=0}^{n-2} \frac{\psi^{ ( l+1 )} ( \delta_{2} )}{l! ( l+2 )} \frac{ ( z-\delta_{2} ) ^{l+2}}{\delta_{2}-\delta _{1}} \\ &\quad {} +\frac{1}{ ( n-1 ) !} \int_{\delta_{1}}^{\delta _{2}}R_{n} ( z,\xi ) \psi^{ ( n )} ( \xi ) \,d\xi, \end{aligned}$$

*where*

4 $$ R_{n} ( z,\xi ) = \textstyle\begin{cases} -\frac{ ( z-\xi ) ^{n}}{n ( \delta_{2}-\delta_{1} )} +\frac{z-\delta_{1}}{\delta_{2}-\delta_{1}} (z-\xi ) ^{n-1}, & \delta_{1}\leq\xi\leq z,\\ -\frac{ ( z-\xi ) ^{n}}{n ( \delta_{2}-\delta_{1} )} +\frac{z-\delta_{2}}{\delta_{2}-\delta_{1} } (z-\xi ) ^{n-1}, & z< \xi\leq\delta_{2}. \end{cases} $$

In the case $n=1$, the sum $\sum_{l=0}^{n-2}\cdots$ is empty, so identity (3) reduces to the well-known Montgomery identity (see, for instance, [11])

$$\psi ( z ) =\frac{1}{\delta_{2}-\delta_{1}} \int_{\delta _{1}}^{\delta_{2}}\psi ( \xi ) \,d\xi+ \int _{\delta_{1}}^{\delta_{2}}P ( z,s ) \psi^{\prime} ( \xi ) \,d\xi, $$

where $P ( z,\xi ) $ is the Peano kernel defined by

$$P ( z,\xi ) = \textstyle\begin{cases} \frac{\xi-\delta_{1}}{\delta_{2}-\delta_{1}}, & \delta_{1}\leq\xi\leq z,\\ \frac{\xi-\delta_{2}}{\delta_{2}-\delta_{1}}, & z< \xi\leq\delta_{2}. \end{cases} $$

### Remark 2

It is important to note that $R_{n}\ge0$ for even *n*, as

$$ R_{n} ( z,\xi ) = \textstyle\begin{cases} \frac{ ( z-\xi ) ^{n-1}}{n ( \delta_{2}-\delta _{1} )} (n(z-\delta_{1})-(z-\xi) ), & \delta_{1}\leq\xi \leq z,\\ \frac{(-1)^{n} ( \xi-z ) ^{n-1}}{n ( \delta_{2}-\delta _{1} )} (n(\delta_{2}-z)-(\xi-z) ), & z< \xi\leq\delta_{2}. \end{cases} $$

Now $\delta_{1}\leq\xi\leq z \Leftrightarrow z-\delta_{1}\ge z-\xi$. As $n>1$, so $n(z-\delta_{1})-(z-\xi)\ge0$.

Also $z<\xi\leq\delta_{2} \Leftrightarrow\delta_{2}-z \ge\xi-z$. As $n>1$, so $n(\delta_{2}-z)-(\xi-z)\ge0$.

The complete reference concerning the Abel-Gontscharoff polynomial and theorem for ‘two-point right focal’ problem is given in [12]. As a special choice for $n=2$, the Abel-Gontscharoff polynomial for ‘two-point right focal’ interpolating polynomial can be given as

5 $$ \psi(z)=\psi(\delta_{1})+(z-\delta_{1}) \psi'(\delta_{2})+ \int _{\delta_{1}}^{\delta_{2}}{G_{\Lambda,2}(z,w) \psi''(w)}\,dw, $$

where $G_{\Lambda,2}(z, w) $ is the Green’s function for ‘two-point right focal problem’ given as

6 $$ G_{1}(z,w)=G_{\Lambda,2}(z, w) = \textstyle\begin{cases} \delta_{1}-w, & \delta_{1}\le w \le z,\\ \delta_{1}-z, & z\le w \le\delta_{2}. \end{cases} $$

In the next section, we present our main results by introducing some new types of Green’s functions.

## Main results

We start this section by our nice observation about Abel-Gontscharoff identity (5) and the related Green’s function for ‘two-point right focal problem’. Therefore, keeping in view the Abel-Gontscharoff Green’s function for ‘two-point right focal problem’, we would like to introduce some new types of Green’s functions $G_{k}:[\delta_{1},\delta_{2}]\times[\delta_{1},\delta_{2}]\rightarrow \mathbb {R}$, ($k=2,3,4$) defined as

7 $$\begin{aligned}& G_{2}(z,w) = \textstyle\begin{cases} z-\delta_{2}, & \delta_{1}\le w \le z,\\ w-\delta_{2}, & z\le w \le\delta_{2}. \end{cases}\displaystyle \end{aligned}$$

8 $$\begin{aligned}& G_{3}(z,w) = \textstyle\begin{cases} z-\delta_{1}, & \delta_{1}\le w \le z,\\ w-\delta_{1}, & z\le w \le\delta_{2}. \end{cases}\displaystyle \end{aligned}$$

9 $$\begin{aligned}& G_{4}(z,w) = \textstyle\begin{cases} \delta_{2}-w, & \delta_{1}\le w \le z,\\ \delta_{2}-z, & z\le w \le\delta_{2}. \end{cases}\displaystyle \end{aligned}$$

**Figure 1:**
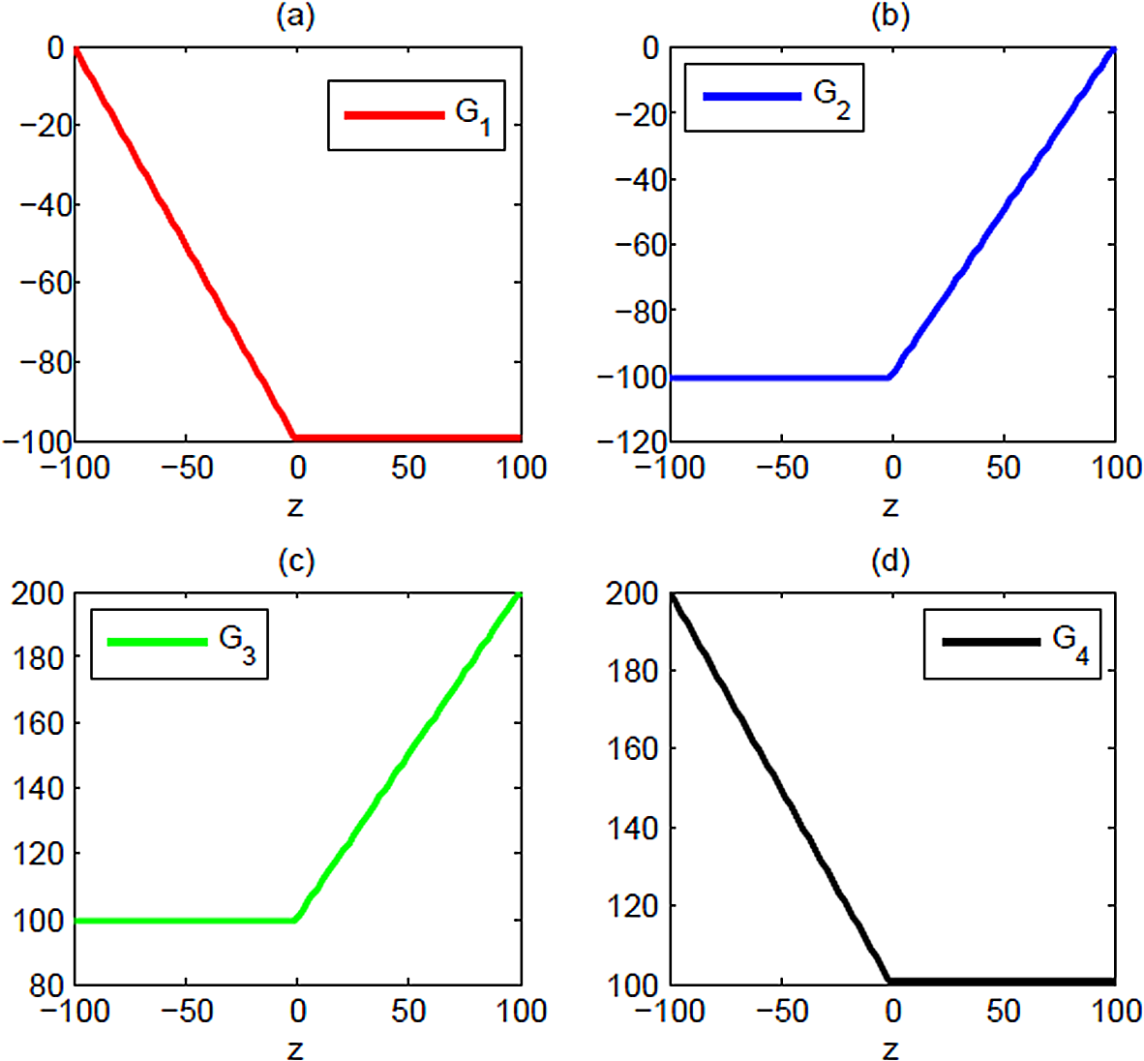
**Graph of Green’s functions for fixed** ***w*** **.**

The graphical representations of $G_{k}$, $k=1,2,3,4$, are depicted in Figure 1 which shows that all four Green’s functions are continuous and symmetric. Moreover, all functions are convex with respect to both the variables z and w. These new Green’s functions enable us to introduce some new identities, stated in the form of the following lemma.

### Lemma 1

*Let* $\psi:[\delta_{1},\delta_{2}]\to \mathbb {R}$ *be a twice differentiable function and* $G_{k}$ ($k=1,2,3,4$) *be the new Green’s functions defined above*. *Then along with* (5) *the following identities hold*:

10 $$\begin{aligned}& \psi(z)=\psi(\delta_{2})+(\delta_{2}-z) \psi'(\delta_{1})+ \int _{\delta_{1}}^{\delta_{2}}{G_{2}(z,w) \psi''(w)}\,dw, \end{aligned}$$

11 $$\begin{aligned}& \psi(z)=\psi(\delta_{2})-(\delta_{2}- \delta_{1})\psi'(\delta _{2})+(z- \delta_{1})\psi'(\delta_{1})+ \int_{\delta_{1}}^{\delta _{2}}{G_{3}(z,w) \psi''(w)}\,dw, \end{aligned}$$

12 $$\begin{aligned}& \psi(z)=\psi(\delta_{1})+(\delta_{2}- \delta_{1})\psi'(\delta _{1})-( \delta_{2}-z)\psi'(\delta_{2})+ \int_{\delta_{1}}^{\delta _{2}}{G_{4}(z,w) \psi''(w)}\,dw. \end{aligned}$$

### Proof

We can give the proofs of the above identities by following the same integrating scheme. Therefore we would like to give the proof of (12) only.

As

$$\begin{aligned} &\int_{\delta_{1}}^{\delta_{2}}{G_{4}(z, w) \psi''(w)}\,dw\\ &\quad = \int_{\delta _{1}}^{z}G_{4}(z,w) \psi''(w)\,dw+ \int_{z}^{\delta_{2}}G_{4}(z,w) \psi''(w)\,dw \\ &\quad = \int_{\delta_{1}}^{z}(\delta_{2}-w) \psi''(w)\,dw+ \int_{z}^{\delta _{2}}(\delta_{2}-z) \psi''(w)\,dw \\ &\quad =(\delta_{2}-w)\psi'(w)\mid_{\delta_{1}}^{z}- \int_{\delta _{1}}^{z}-1.\psi'(w)\,dw+( \delta_{2}-z)\bigl[\psi'(\delta_{2})- \psi'(z)\bigr] \\ &\quad =(\delta_{2}-z)\psi'(z)-(\delta_{2}- \delta_{1})\psi'(\delta_{1})+\psi (z)-\psi( \delta_{1})+(\delta_{2}-z)\psi'( \delta_{2})-(\delta_{2}-z)\psi '(z) \\ &\quad =(\delta_{2}-z)\psi'(\delta_{2})-( \delta_{2}-\delta_{1})\psi'(\delta _{1})-\psi(\delta_{1})+\psi(z). \end{aligned}$$

Now by simplifying terms, we will get our identity (12). □

### Remark 3

Lemma 1 gives another proof of the special case of Abel-Gontscharoff identity (5). $G_{3}$ and $G_{4}$ are new Green’s functions, but the results are not so simple as in other two cases.

The inequality of Popoviciu, which was improved by Vasić and Stanković [1], is generalized by using the above new Green’s functions. In Theorem 1 we have that $q_{i}$ ($i=1,\ldots,s$) are positive real numbers. Now we give the generalization of that result for real values of $q_{i}$ ($i=1,\ldots,s$) with $\sum_{i=1}^{s} q_{i} =1$ using the new Green’s functions $G_{k}$, $k=1,2,3,4$, as defined in Lemma 1.

### Theorem 3

*Let* $[\delta_{1},\delta_{2}]$ *be an interval in* $\mathbb {R}$, *for integers* $m\geq3$, $2\leq s\leq m-1$, *consider the tuples* $\mathbf{z} \in [\delta_{1},\delta_{2}]^{m}$, **q** *be a real* *m*-*tuple such that* $\sum_{j = 1}^{s} {{q_{{i_{j}}}}} \ne0$ *for any* ${1 \le{i_{1}} < \cdots < {i_{s}} \le m}$ *and* $\sum_{i = 1}^{m} {{q_{i}}} = 1$. *Also*, *let* $\frac{{\sum_{j = 1}^{s} {{q_{{i_{j}}}}{z_{{i_{j}}}}}} }{{\sum_{j = 1}^{s} {{q_{{i_{j}}}}}} } \in[\delta_{1},\delta_{2} ]$ *for any* ${1 \le{i_{1}} < \cdots < {i_{s}} \le m}$. *Then the following statements are equivalent*:

(i) *For every continuous convex function* $\psi:[\delta _{1},\delta_{2}]\rightarrow \mathbb {R}$,
  13 $$ {\psi_{s,m}}(\mathbf{z},\mathbf{q}) \le\frac{{m - s}}{{m - 1}} {\psi _{1,m}}(\mathbf{z},\mathbf{q}) + \frac{{s - 1}}{{m - 1}}{\psi _{m,m}}(\mathbf{z},\mathbf{q}) $$
  *holds*, *where*
  $$ {\psi_{s,m}}(\mathbf{z},\mathbf{q}): = \frac{1}{{C_{s - 1}^{m - 1}}}\sum _{1 \le{i_{1}} < \cdots < {i_{s}} \le m} { \Biggl( {\sum_{j = 1}^{s} {{q_{{i_{j}}}}}} \Biggr)\psi \biggl( {\frac{{\sum_{j = 1}^{s} {{q_{{i_{j}}}}{z_{{i_{j}}}}}} }{{\sum_{j = 1}^{s} {{q_{{i_{j}}}}}} }} \biggr)}; $$
(ii) *For all* $w\in[\delta_{1},\delta_{2}]$ *and* $k=1,2,3,4$,
  14 $$ {G_{s,m}^{k}}(\mathbf{z},w;\mathbf{q}) \le \frac{{m - s}}{{m - 1}}{G_{1,m}^{k}}(\mathbf{z},w;\mathbf{q}) + \frac{{s - 1}}{{m - 1}}{G^{k}_{m,m}}(\mathbf{z},w;\mathbf{q}), $$
  *where*
  $$\begin{gathered} {G_{s,m}^{k}}(\mathbf{z},w; \mathbf{q}) \\ \quad: = \frac{1}{{ C_{s - 1}^{m - 1}}}\sum_{1 \le{i_{1}} < \cdots < {i_{s}} \le m} { \Biggl( { \sum_{j = 1}^{s} {{q_{{i_{j}}}}}} \Biggr)G_{k} \biggl( {\frac{{\sum_{j = 1}^{s} {{q_{{i_{j}}}}{z_{{i_{j}}}}}} }{{\sum_{j = 1}^{s} {{q_{{i_{j}}}}}} },w} \biggr)};\quad2 \le s \le m, \end{gathered} $$
  *for the functions* $G_{k}:[\delta_{1},\delta_{2}]\times[\delta_{1},\delta _{2}]\rightarrow \mathbb {R}$.

*Moreover*, *the statements* (i) *and* (ii) *are also equivalent if we change the sign of the inequality in both* (13) *and* (14).

### Proof

$\mbox{(i)}\Rightarrow\mbox{(ii)}$: Let (i) be valid. Fix $k=1,2,3,4$. Then as the functions for all *k* $G_{k}(\cdot, w)$ ($w \in[\delta_{1},\delta_{2}]$) are also continuous and convex, it follows that for these functions (13) also holds for each fix *k*, i.e., (14) is valid.

$\mbox{(ii)}\Rightarrow\mbox{(i)}$: Let $\psi:[\delta_{1},\delta_{2}]\rightarrow \mathbb {R}$ be a convex function, $\psi\in C^{2}([\delta_{1},\delta_{2}])$ and (ii) holds. Then, by Lemma 1, we can represent a function *ψ* in the form (5), (10), (11) and (12) for their respective fixed *k*. Now, by means of some simple calculations, we can write

15 $$ \begin{gathered}[b] \frac{{m - s}}{{m - 1}} { \psi_{1,m}}(\mathbf{z},\mathbf{q}) + \frac{{s - 1}}{{m - 1}}{ \psi_{m,m}}(\mathbf{z},\mathbf{q}) - {\psi_{s,m}}(\mathbf{z}, \mathbf{q}) \\ \quad = \int_{\delta_{1}} ^{\delta_{2}} { \biggl( \frac{{m - s}}{{m - 1}}{{G_{1,m}^{k}}(\mathbf{z},w;\mathbf{q}) + \frac{{s - 1}}{{m - 1}}{G^{k}_{m,m}}(\mathbf{z},w;\mathbf{q}) - {G_{s,m}^{k}}(\mathbf{z},w;\mathbf{q})} \biggr)} \psi''(w)\,dw. \end{gathered} $$

By the convexity of *ψ*, we have $\psi''(w)\ge0$ for all $w\in [\delta_{1},\delta_{2}]$. Hence, if for every $w\in[\delta_{1},\delta _{2}]$, (14) is valid for each $k=1,2,3,4$, then it follows that for every convex function $\psi:[\delta_{1},\delta_{2}]\rightarrow \mathbb {R}$, with $\psi\in C^{2}([\delta_{1},\delta_{2}])$, (13) is valid.

Here we can eliminate the differentiability condition due to the fact that it is possible to approximate uniformly a continuous convex function by convex polynomials (see [2], p.172).

Analogous to the above proof, we can give the proof of the last part of our theorem. □

Next we formulate generalized identities with the help of identities defined in Lemma 1 and Fink’s identity.

### Theorem 4

*Let all the assumptions of Theorem* 2 *be valid with* $n>2$, *and let* $m, s\in{\mathbb{N}}$, $m\geq3$, $2\leq s\leq m-1$, $\mathbf{z} \in[\delta_{1},\delta_{2}]^{m}$, **q** *be a real* *m*-*tuple such that* $\sum_{j = 1}^{s} {{q_{{i_{j}}}}} \ne0$ *for any* ${1 \le{i_{1}} < \cdots < {i_{s}} \le m}$ *and* $\sum_{i = 1}^{m} {{q_{i}}} = 1$. *Also let* $\frac{{\sum_{j = 1}^{s} {{q_{{i_{j}}}}{z_{{i_{j}}}}}} }{{\sum_{j = 1}^{s} {{q_{{i_{j}}}}}} } \in[\delta_{1},\delta_{2} ]$ *for any* ${1 \le{i_{1}} < \cdots < {i_{s}} \le m}$ *with* $R_{n}( \cdot,v)$ *and* $G_{k}(\cdot, w)$, ($k=1,2,3,4$) *be the same as defined in* (4) *and Lemma* 1, *respectively*. *Then we have the following new identities for* $k=1,2,3,4$:

16 $$ \begin{aligned}[b] &\operatorname{POP}\bigl[\mathbf{z}, \mathbf{q};\psi(z)\bigr]\\ &\quad = \biggl(\frac {{\psi'(\delta_{1} ) - \psi'(\delta_{2} )}}{{\delta_{2} - \delta_{1} }} \biggr) \int_{\delta_{1}}^{\delta_{2}} {\operatorname{POP}\bigl[\mathbf{z}, \mathbf{q};G_{k}(z,w)\bigr]}\,dw \\ &\qquad{} + \frac{1}{{\delta_{2} - \delta_{1}} } \int_{\delta_{1}} ^{\delta_{2}} \operatorname{POP}\bigl[\mathbf{z}, \mathbf{q};G_{k}(z,w)\bigr] \\ &\qquad {}\times\Biggl(\sum_{l = 2}^{n - 1} \frac{l}{{(l - 1)!}} \bigl( {\psi^{ ( l )}} ( \delta_{1} ){{ ( {w - \delta_{1}} )}^{l - 1}}- {\psi^{ ( l )}} ( \delta_{2} ){{ ( {w - \delta_{2}} )}^{l - 1}} \bigr) \Biggr) \,dw \\ &\qquad{} + \frac{1}{{ ( {n - 3} )!}} \int_{\delta_{1}} ^{\delta_{2}} {{\psi^{(n)}}} (v) \biggl( \int_{\delta_{1}} ^{\delta_{2}} {\operatorname{POP}\bigl[\mathbf{z}, \mathbf{q};G_{k}(z,w)\bigr]} {{\tilde{R}}_{n - 2}} ( {w,v} )\,dw \biggr)\,dv, \end{aligned} $$

*where*

$$\tilde{R}_{n-2} ( {w,v} )= \textstyle\begin{cases} {\frac{1}{{\delta_{2} - \delta_{1}} } [ {\frac{{{{ ( {w - v} )}^{n - 2}}}}{{(n - 2)}} + ( {w - \delta_{1}} ){{ ( {w - v} )}^{n - 3}}} ],}&{\delta_{1} \le v \le w,}\\ {\frac{1}{{\delta_{2} - \delta_{1}} } [ {\frac{{{{ ( {w - v} )}^{n - 2}}}}{{(n - 2)}} + ( {w - \delta_{2}} ){{ ( {w - v} )}^{n - 3}}} ],}&{w < v \le\delta_{2},} \end{cases} $$

*and*

17 $$ \begin{aligned}[b] &\operatorname{POP}\bigl[\mathbf{z}, \mathbf{q};\psi(z)\bigr] \\ &\quad = \biggl( {\frac {{\psi'(\delta_{2} ) - \psi'(\delta_{1} )}}{{\delta_{2} - \delta_{1}} }} \biggr) \int_{\delta_{1}} ^{\delta_{2}} {\operatorname{POP}\bigl[\mathbf{z}, \mathbf{q};G_{k}(z,w)\bigr]} \,dw \\ &\qquad{} + \frac{1}{{\delta_{2} - \delta_{1}} } \int_{\delta_{1}} ^{\delta_{2}} \operatorname{POP}\bigl[\mathbf{z}, \mathbf{q};G_{k}(z,w)\bigr]\\ &\qquad{}\times \Biggl({\sum_{l = 3}^{n - 1} {\frac{{{\psi^{ ( l )}} (\delta_{1} ){{ ( {w - \delta_{1}} )}^{l - 1}} - {\psi ^{ ( l )}} ( \delta_{2} ){{ ( {w - \delta _{2}} )}^{l - 1}}}}{{(l - 3)!(l - 1)}}}} \Biggr) \,dw \\ &\qquad{} + \frac{1}{{ ( {n - 3} )!}} \int_{\delta_{1}} ^{\delta_{2}} {{\psi^{ ( n )}} ( v ) \biggl({ \int_{\delta_{1}} ^{\delta_{2}} {\operatorname{POP}\bigl[\mathbf{z}, \mathbf{q};G_{k}(z,w)\bigr]} {R_{n - 2}}(w,v)\,dw} \biggr)\,dv}. \end{aligned} $$

### Proof

Fix $k=1,2,3,4$. Applying Popoviciu’s functional (2) to identities (5), (10), (11), (12) along with their respective new Green’s functions and following the properties of $\operatorname{POP}[\mathbf{z},\mathbf{q};\cdot]$, we get

18 $$ \operatorname{POP}\bigl[\mathbf{z},\mathbf{q};\psi(z)\bigr]= \int_{\delta _{1}} ^{\delta_{2}} {\operatorname{POP}\bigl[\mathbf{z}, \mathbf{q};G_{k}(z, w)\bigr]} \psi''(w)\,dw. $$

Differentiating (3) twice with respect to the first variable, we have

19 $$ \begin{aligned}[b] \psi'' ( w ) &= \frac{{\psi' ( \delta_{1} ) - \psi' ( \delta_{2} )}}{{\delta_{2} - \delta_{1}} } \\ &\quad{} + \sum_{l = 2}^{n - 1} { \biggl( { \frac{l}{{(l - 1)!}}} \biggr)} \biggl( {\frac{{{\psi^{ ( l )}} ( \delta _{1} ){{ ( {w - \delta_{1}} )}^{l - 1}} - {\psi ^{ ( l )}} ( \delta_{2} ){{ ( {w - \delta _{2}} )}^{l - 1}}}}{{\delta_{2} - \delta_{1}} }} \biggr) \\ &\quad{} + \frac{1}{{ ( {n - 3} )!}} \int_{\delta_{1}} ^{\delta_{2}} {{{\tilde{R}}_{n - 2}}} ( {w,v} ){\psi^{ ( n )}} ( v )\,dv. \end{aligned} $$

Using (19) in (18), we get

$$\begin{aligned} \operatorname{POP}\bigl[\mathbf{z},\mathbf{q};\psi(z) \bigr] &= \biggl(\frac {{\psi'(\delta_{1} ) - \psi'(\delta_{2} )}}{{\delta_{2} - \delta_{1} }} \biggr) \int_{\delta_{1}}^{\delta_{2}} {\operatorname{POP}\bigl[\mathbf{z}, \mathbf{q};G_{k}(z,w)\bigr]}\,dw \\ &\quad{} + \sum_{l = 2}^{n - 1} { \frac{l}{{(l - 1)!}}} \int_{\delta _{1}} ^{\delta_{2}} \operatorname{POP}\bigl[\mathbf{z}, \mathbf{q};G_{k}(z,w)\bigr]\\ &\quad {}\times \biggl( {\frac{{{\psi^{ ( l )}} (\delta_{1} ){{ ( {w - \delta_{1}} )}^{l - 1}} - {\psi ^{ ( l )}} ( \delta_{2} ){{ ( {w - \delta _{2}} )}^{l - 1}}}}{{\delta_{2} - \delta_{1}} }} \biggr) \,dw \\ &\quad{} + \frac{1}{{ ( {n - 3} )!}} \int_{\delta_{1}} ^{\delta_{2}} {\mathbf{P}\bigl(\mathbf{x}, \mathbf{p};G_{k}(z,w)\bigr)} \biggl( \int_{\delta _{1}} ^{\delta_{2}} {{{\tilde{R}}_{n - 2}}} ( {w,v} ){\psi ^{ ( n )}} ( v )\,dv\biggr)\,dw. \end{aligned} $$

By executing Fubini’s theorem in the last term, we have (16) respectively for $k=1,2,3,4$.

Next, using formula (3) on the function $\psi''$, replacing *n* by $n-2$ ($n\geq3$) and rearranging the indices, we have

20 $$ \begin{aligned}[b] \psi'' ( w ) &= \biggl( {\frac{{\psi'(\delta_{2} ) - \psi '(\delta_{1} )}}{{\delta_{2} - \delta_{1}} }} \biggr) \\ &\quad{} + \sum_{l = 3}^{n - 1} { \biggl( { \frac{1}{{(l-3)!(l -1)}}} \biggr)} \biggl( {\frac{{{\psi^{ ( {l} )}} ( \delta _{1} ){{ ( {w - \delta_{1}} )}^{l-1}} - {\psi^{ ( {l} )}} ( \delta_{2} ){{ ( {w - \delta_{2}} )}^{l-1}}}}{{\delta_{2} - \delta_{1}} }} \biggr) \\ &\quad{} + \frac{1}{{ ( {n - 3} )!}} \int_{\delta_{1}} ^{\delta_{2}} {{R_{n - 2}}(w,v){ \psi^{ ( n )}} ( v )\,dv}. \end{aligned} $$

Similarly, using (20) in (18) and employing Fubini’s theorem, we get (17) respectively for $k=1,2,3,4$. □

As an application of the above obtained identities, the next theorem gives artistic generalization of Popoviciu-type inequalities for *n*-convex functions involving new Green’s functions.

### Theorem 5

*Let all the assumptions of Theorem* 4 *be satisfied and* $n\ge 3$. *Also let* *ψ* *be an* *n*-*convex function such that* $\psi ^{(n-1)}$ *is absolutely continuous*. *Then we have the following two results*:

*If*

21 $$ \int_{\delta_{1}} ^{\delta_{2}} {\operatorname{POP}\bigl[\mathbf{z}, \mathbf{q};G_{k}(z,w)\bigr]} {{\tilde{R}}_{n - 2}} ( {w,v} )\,dw \ge0, \quad v\in[\delta_{1},\delta_{2}] $$

*for* $k=1,2,3,4$, *then*

22 $$ \begin{aligned}[b] \operatorname{POP}\bigl[\mathbf{z}, \mathbf{q};\psi(z)\bigr] &\ge \biggl(\frac {{\psi'(\delta_{1} ) - \psi'(\delta_{2} )}}{{\delta_{2} - \delta_{1} }} \biggr) \int_{\delta_{1}}^{\delta_{2}} {\operatorname{POP}\bigl[\mathbf{z}, \mathbf{q};G_{k}(z,w)\bigr]}\,dw \\ &\quad{} + \frac{1}{{\delta_{2} - \delta_{1}} } \int_{\delta_{1}} ^{\delta_{2}} \operatorname{POP}\bigl[\mathbf{z}, \mathbf{q};G_{k}(z,w)\bigr] \\ &\quad{}\times\Biggl({\sum_{l = 2}^{n - 1} {\frac{l}{{(l - 1)!}} \bigl( {{\psi^{ ( l )}} ( \delta_{1} ){{ ( {w - \delta_{1}} )}^{l - 1}} - {\psi^{ ( l )}} ( \delta_{2} ){{ ( {w - \delta_{2}} )}^{l - 1}}} \bigr)}} \Biggr) \,dw; \end{aligned} $$

*and if*

23 $$ \int_{\delta_{1}} ^{\delta_{2}} {\operatorname{POP}\bigl[\mathbf{z}, \mathbf{q};G_{k}(z,w)\bigr]} {R_{n - 2}} ( {w,v} )\,dw\ge0, \quad v\in [\delta_{1},\delta_{2}] $$

*for* $k=1,2,3,4$, *then*

24 $$ \begin{aligned}[b] \operatorname{POP}\bigl[\mathbf{z}, \mathbf{q};\psi(z)\bigr] &\ge \biggl( {\frac {{\psi'(\delta_{2} ) - \psi'(\delta_{1} )}}{{\delta_{2} - \delta_{1}} }} \biggr) \int_{\delta_{1}} ^{\delta_{2}} {\operatorname{POP}\bigl[\mathbf{z}, \mathbf{q};G_{k}(z,w)\bigr]} \,dw \\ &\quad{} + \frac{1}{{\delta_{2} - \delta_{1}} } \int_{\delta_{1}} ^{\delta_{2}} \operatorname{POP}\bigl[\mathbf{z}, \mathbf{q};G_{k}(z,w)\bigr]\\ &\quad{}\times \Biggl({\sum_{l = 3}^{n - 1} {\frac{{{\psi^{ ( l )}} (\delta_{1} ){{ ( {w - \delta_{1}} )}^{l - 1}} - {\psi ^{ ( l )}} ( \delta_{2} ){{ ( {w - \delta _{2}} )}^{l - 1}}}}{{(l - 3)!(l - 1)}}}} \Biggr) \,dw. \end{aligned} $$

### Proof

Fix $k=1,2,3,4$. Since $\psi^{(n-1)}$ is absolutely continuous on $[\delta_{1},\delta_{2}]$, $\psi^{(n)}$ exists almost everywhere. As *ψ* is *n*-convex, so $\psi^{(n)}(z)\ge0$ for all $z\in[\delta _{1},\delta_{2}]$ (see [2], p.16). Hence we can apply Theorem 4 to obtain (22) and (24) respectively. □

### Remark 4

Inequalities (22) and (23) hold in reverse directions if the inequalities in (21) and (23) are reversed.

Now we state the final result of this section in the form of the following theorem.

### Theorem 6

*In addition to the assumptions of Theorem* 4, *let* $\mathbf {q}=(q_{1},\ldots,q_{m})$ *be a positive* *m*-*tuple such that* $\sum_{i = 1}^{m} {{q_{i}}} = 1$, *and* $\psi:[\delta_{1},\delta_{2}] \to \mathbb {R}$ *be an* *n*-*convex function*.

(i) *For fixed* $k=1,2,3,4$, *inequalities* (22) *and* (24) *hold provided that* *n* *is even and* ($n\ge4$).
(ii) *For fixed* $k=1,2,3,4$, *let inequality* (22) *be satisfied and*
  25 $$ \sum_{l = 1}^{n - 1} { \frac{l}{{(l - 1)!}} \bigl( {{\psi^{ ( l )}} ( \delta_{1} ){{ ( {w - \delta_{1}} )}^{l - 1}} - {\psi^{ ( l )}} ( \delta_{2} ){{ ( {w - \delta_{2}} )}^{l - 1}}} \bigr)} \ge0;\quad \forall w \in[\delta_{1}, \delta_{2}], $$
  *OR* (24) *be satisfied and*
  26 $$ \begin{aligned}[b] &\psi'(\delta_{2} ) - \psi'( \delta_{1} )\\ &\quad {} + \sum_{l = 3}^{n - 1} { \frac {{{\psi^{ ( l )}} ( \delta_{1} ){{ ( {w - \delta_{1}} )}^{l - 1}} - {\psi^{ ( l )}} (\delta_{2} ){{ ( {w - \delta_{2}} )}^{l - 1}}}}{{(l - 3)!(l - 1)}}} \ge0;\quad\forall w \in[\delta_{1}, \delta_{2}]. \end{aligned} $$
  *Then we have*
  27 $$ \operatorname{POP}\bigl[\mathbf{z},\mathbf{q};\psi(z)\bigr]\ge0. $$

### Proof

It is clear from Figure 1 that Green’s function $G_{k}(z, w)$ is convex for all $k=1,2,3,4$, and the weights are assumed to be positive. Therefore, applying Theorem 1 and taking into account Remark 1, we can obtain $\operatorname{POP}[\mathbf{z},\mathbf{q};G_{k}(z, w)]\ge0$ for all $k=1,2,3,4$.

(i) $\tilde{R}_{n-2} ( {w,v} )\ge0$ and $R_{n-2} ( {w,v} )\ge0$ for $n=4,6,\ldots$ , so (21) and (23) hold. As *ψ* is *n*-convex, hence by following Theorem 5, we obtain (22) and (24).
(ii) Using (25) in (22) and (26) in (24), (27) is established for all $k=1,2,3,4$. □

## Applications to $(n+1)$-convex functions at a point

In the present section we give related results for the class of $(n+1)$-convex functions at a point introduced in [4].

### Definition 1

Let $I\subseteq \mathbb {R}$ be an interval, $c\in I^{o}$ and $n\in\mathbb {N}$. A function $\psi:I \rightarrow \mathbb {R}$ is said to be $(n+1)$-convex at point *c* if there exists a constant $Z_{c}$ such that the function

28 $$ \Psi(z)=\psi(z)-\frac{Z_{c}}{n!}z^{n} $$

is *n*-concave on $I \cap(-\infty,c]$ and *n*-convex on $I \cap [c,\infty)$. A function *ψ* is said to be $(n+1)$-concave at point *c* if the function −*ψ* is $(n+1)$-convex at point *c*.

A function is $(n+1)$-convex on an interval if and only if it is $(n+1)$-convex at every point of the interval (see [4]). Pečarić, Praljak and Witkowski in [4] studied necessary and sufficient conditions on two linear functionals $\Omega:C([\delta _{1},c])\rightarrow \mathbb {R}$ and $\Lambda:C([c, \delta_{2} ])\rightarrow \mathbb {R}$ so that the inequality $\Omega(\psi)\le\Lambda(\psi)$ holds for every function *ψ* that is $(n+1)$-convex at point *c*. In the present section we give inequalities of such type for the particular linear functionals obtained from the inequalities in the previous section. Let $\sigma_{i}$ denote the monomials $\sigma_{i}(z)=z^{i}$, $i\in\mathbb {N}_{0}$. For the rest of this section, $\Omega_{k}(\psi)$ and $\Lambda _{k}(\psi)$ for fixed $k=1,2,3,4$ will denote the linear functionals obtained as the difference of the L.H.S. and R.H.S. of inequality (22), applied to the intervals $[\delta_{1},c]$ and $[c,\delta_{2}]$, respectively, i.e., for $\mathbf{z}\in[\delta _{1},c]^{m}$, $\mathbf{q}\in \mathbb {R}^{m}$, $\mathbf{y}\in[c,\delta_{2}]^{\bar{m}}$ and $\bar{\mathbf{q}}\in \mathbb {R}^{\bar{m}}$, let

29 $$\begin{aligned}& \begin{aligned}[b] \Omega_{k}(\psi)&: ={ \operatorname{POP}\bigl[\mathbf{z},\mathbf{q};\psi(z)\bigr] - \biggl( \frac{{\psi'(\delta_{1} ) - \psi'(c )}}{{c - \delta_{1} }} \biggr) \int_{\delta_{1}}^{c} {\operatorname{POP}\bigl[\mathbf{z}, \mathbf{q};G_{k}(z,w)\bigr]}\,dw} \\ &\quad{} + \frac{1}{{c - \delta_{1}} } \int_{\delta_{1}} ^{c} \operatorname{POP}\bigl[\mathbf{z}, \mathbf{q};G_{k}(z,w)\bigr]\\ &\quad{}\times \Biggl( {\sum_{l = 2}^{n - 1} {\frac{l}{{(l - 1)!}} \bigl( {{\psi^{ ( l )}} ( \delta_{1} ){{ ( {w - \delta_{1}} )}^{l - 1}} - {\psi^{ ( l )}} ( c ){{ ( {w - c} )}^{l - 1}}} \bigr)}} \Biggr) \,dw, \end{aligned} \end{aligned}$$

30 $$\begin{aligned}& \begin{aligned}[b] \Lambda_{k}(\psi)&: = { \operatorname{POP}\bigl[\mathbf{y},\bar{\mathbf{q}};\psi(y)\bigr] - \biggl( \frac{{\psi'(c ) - \psi'(\delta_{2} )}}{{\delta_{2} - c} } \biggr) \int_{c}^{\delta_{2}} {\operatorname {POP}\bigl[\mathbf{y}, \bar{\mathbf{q}};G_{k}(y,w)\bigr]}\,dw} \\ &\quad{} + \frac{1}{{\delta_{2} - c} } \int_{c} ^{\delta_{2}} \operatorname{POP}\bigl[\mathbf{y}, \bar{\mathbf{q}};G_{k}(y,w)\bigr]\\ &\quad{}\times \Biggl( {\sum _{l = 2}^{n - 1} {\frac{l}{{(l - 1)!}} \bigl( {{ \psi^{ ( l )}} ( c ){{ ( {w - c} )}^{l - 1}} - {\psi^{ ( l )}} ( \delta_{2} ){{ ( {w - \delta_{2}} )}^{l - 1}}} \bigr)}} \Biggr) \,dw. \end{aligned} \end{aligned}$$

It is important to notify that by introducing new linear functionals for $k=1,2,3,4$, $\Omega_{k}(\psi)$ and $\Lambda_{k}(\psi)$, identity (16) applied to the respective intervals $[\delta_{1},c]$ and $[c,\delta_{2}]$ takes the shape:

31 $$\begin{aligned}& \Omega_{k}(\psi)= \frac{1}{ ( n-3 ) !} \int_{\delta_{1}} ^{c}{ \psi^{ (n )} (v )} \biggl( \int_{\delta _{1}} ^{c} {\operatorname{POP}\bigl[\mathbf{z}, \mathbf{q};G_{k}(z,w)\bigr]} {{\tilde{R}}_{n - 2}} ( {w,v} )\,dw \biggr)\,dv, \end{aligned}$$

32 $$\begin{aligned}& \Lambda_{k}(\psi)= \frac{1}{ ( n-3 ) !} \int_{c} ^{\delta _{2}}{ \psi^{ (n )} (v )} \biggl( \int_{c} ^{\delta_{2}} {\operatorname{POP}\bigl[\mathbf{y}, \bar{\mathbf{q}};G_{k}(y,w)\bigr]} {{\tilde{R}}_{n - 2}} ( {w,v} ) \,dw \biggr)\,dv. \end{aligned}$$

Now we are ready to state the following theorem for inequalities involving $(n+1)$-convex function at a point.

### Theorem 7

*Let* $\mathbf{z}\in[\delta_{1},c]^{m}$, $\mathbf{q}\in \mathbb {R}^{m}$, $\mathbf {y}\in[c,\delta_{2}]^{\bar{m}}$ *and* $\bar{\mathbf{q}}\in \mathbb {R}^{\bar{m}}$ *in such a way that for* $k=1,2,3,4$,

33 $$\begin{aligned}& \int_{\delta_{1}} ^{c} {\operatorname{POP}\bigl[\mathbf{z}, \mathbf{q};G_{k}(z,w)\bigr]} {{\tilde{R}}_{n - 2}} ( {w,v} )\,dw \ge0, \quad v\in[\delta_{1},c], \end{aligned}$$

34 $$\begin{aligned}& \int_{c} ^{\delta_{2}} {\operatorname{POP}\bigl[\mathbf{y}, \bar{\mathbf{q}};G_{k}(y,w)\bigr]} {{\tilde{R}}_{n - 2}} ( {w,v} ) \,dw\ge0, \quad v\in[c,\delta_{2}], \end{aligned}$$

35 $$\begin{aligned}& \begin{gathered}[b] \int_{\delta_{1}} ^{c} \biggl( \int_{\delta_{1}} ^{c} {\operatorname {POP}\bigl[\mathbf{z}, \mathbf{q};G_{k}(z,w)\bigr]} {{\tilde{R}}_{n - 2}} ( {w,v} )\,dw \biggr)\,dv\\ \quad = \int_{c} ^{\delta_{2}} \biggl( \int_{c} ^{\delta _{2}} {\operatorname{POP}\bigl[\mathbf{y}, \bar{\mathbf{q}};G_{k}(y,w)\bigr]} {{\tilde{R}}_{n - 2}} ( {w,v} ) \,dw \biggr)\,dv, \end{gathered} \end{aligned}$$

*where* ${{\tilde{R}}_{n - 2}}( \cdot,\xi)$ *and* $G_{k}(\cdot, w)$, ($k=1,2,3,4$) *be the same as defined in Theorem* 4 *and Lemma* 1, *respectively*, *and let* $\Omega_{k}(\psi)$, $\Lambda_{k}(\psi )$ *be the linear functionals given by* (29) *and* (30). *If* $\psi:[\delta_{1},\delta_{2}]\to \mathbb {R}$ *is* $(n+1)$-*convex at point* *c*, *then we get the monotonicity*

36 $$ \Omega_{k}(\psi)\le\Lambda_{k}(\psi)\quad \textit{for } k=1,2,3,4. $$

*If the inequalities in* (33) *and* (34) *are reversed*, *then* (36) *holds with the reversed sign of inequality*.

### Proof

Using Definition 1, construct the function $\Psi(z)=\psi (z)-\frac{Z_{c}}{n!}\sigma_{n}$ in such a way that the function Ψ is *n*-concave on $[\delta_{1},c]$ and *n*-convex on $[c,\delta_{2}]$. Fix $k=1,2,3,4$, and applying Theorem 5 to Ψ on the interval $[\delta_{1},c]$, we have

37 $$ 0\ge\Omega_{k}(\Psi)=\Omega_{k}(\psi)- \frac{Z_{c}}{n!}\Omega_{k}(\sigma_{n}). $$

Analogously applying Theorem 5 to Ψ on the interval $[c,\delta_{2}]$, we get

38 $$ 0\le\Lambda_{k}(\Psi)=\Lambda_{k}(\psi)- \frac{Z_{c}}{n!}\Lambda _{k}\bigl(\sigma^{n}\bigr). $$

Moreover, identities (33) and (34) applied to the function $\sigma^{n}$ gives

39 $$\begin{aligned}& \Omega_{k}\bigl(\sigma^{n}\bigr)= \bigl(n^{3}-3n^{2}+2n\bigr) \int_{\delta_{1}} ^{c} \biggl( \int _{\delta_{1}} ^{c} {\operatorname{POP}\bigl[\mathbf{z}, \mathbf{q};G_{k}(z,w)\bigr]} {{\tilde{R}}_{n - 2}} ( {w,v} )\,dw \biggr)\,dv, \end{aligned}$$

40 $$\begin{aligned}& \Lambda_{k}\bigl(\sigma^{n}\bigr)= \bigl(n^{3}-3n^{2}+2n\bigr) \int_{c} ^{\delta_{2}} \biggl( \int _{c} ^{\delta_{2}} {\operatorname{POP}\bigl[\mathbf{y}, \bar{\mathbf{q}};G_{k}(y,w)\bigr]} {{\tilde{R}}_{n - 2}} ( {w,v} ) \,dw \biggr)\,dv. \end{aligned}$$

Therefore assumption (35) is equivalent to

$$\Omega_{k}\bigl(\sigma^{n}\bigr)= \Lambda_{k} \bigl(\sigma^{n}\bigr). $$

So, from (37) and (38), we obtain the desired result. □

### Remark 5

In the proof of Theorem 7, we have shown that for $k=1,2,3,4$,

41 $$ \Omega_{k}(\psi)\le\frac{Z_{c}}{n!} \Omega_{k}\bigl(\sigma^{n}\bigr)=\frac {Z_{c}}{n!} \Lambda_{k}\bigl(\sigma^{n}\bigr)\le\Lambda_{k}( \psi). $$

More importantly, inequality (36) still holds if we replace assumption (35) with the weaker assumption that is $Z_{c} (\Lambda_{k}(\sigma^{n})-\Omega_{k}(\sigma^{n}) )\ge0$.

We conclude this section by adding the following remark.

### Remark 6

Similar results can also be given by constructing linear functionals from inequality (24) involving new Green’s functions $G_{k}$ for $k=1,2,3,4$.

## New upper bounds of Grüss and Ostrowski type for generalized identities

In the present section we use Čebyšev’s functional defined for Lebesgue integrable functions $\mathbb{F}_{1},\mathbb{F}_{2}: [\delta _{1},\delta_{2}] \to \mathbb {R}$ as

$$\mathbb{C}(\mathbb{F}_{1},\mathbb{F}_{2})= \frac{1}{\delta_{2}-\delta _{1}} \int_{\delta_{1}}^{\delta_{2}}{\mathbb{F}_{1}(\xi) \mathbb{F}_{2}(\xi )}\,d\xi-\frac{1}{\delta_{2}-\delta_{1}} \int_{\delta_{1}}^{\delta _{2}}{\mathbb{F}_{1}(\xi)}\,d\xi. \frac{1}{\delta_{2}-\delta_{1}} \int _{\delta_{1}}^{\delta_{2}}{\mathbb{F}_{2}(\xi)}\,d\xi, $$

to construct some new upper bounds.

The following inequalities of Grüss type were given in [13].

### Theorem 8

*Let* $\mathbb{F}_{1}\in L[\delta_{1}, \delta_{2}]$ *and* $\mathbb{F}_{2}: [\delta_{1},\delta_{2}] \to \mathbb {R}$ *be an absolutely continuous function along with* $(\cdot-\delta_{1})(\delta_{2}-\cdot)[\mathbb{F}_{2}']^{2}\in L[\delta _{1}, \delta_{2}]$. *Then the inequality*

42 $$ \bigl\vert \mathbb{C}(\mathbb{F}_{1}, \mathbb{F}_{2}) \bigr\vert \le \frac{1}{\sqrt {2}} \biggl[ \frac{\mathbb{C}(\mathbb{F}_{1},\mathbb{F}_{1})}{\delta _{2}-\delta_{1} } \biggr]^{\frac{1}{2}} \biggl( \int_{\delta_{1}}^{\delta_{2}}{(z-\delta _{1}) ( \delta_{2}-z)\bigl[\mathbb{F}_{2}'(z) \bigr]^{2}}\,dz \biggr)^{\frac{1}{2}} $$

*holds with* $\frac{1}{\sqrt{2}}$ *being the best possible constant*.

### Theorem 9

*Let* $\mathbb{F}_{1}: [\delta_{1},\delta_{2}] \to \mathbb {R}$ *be an absolutely continuous function with* $\mathbb{F}_{1}'\in L_{\infty}[\delta_{1}, \delta_{2}]$ *and* $\mathbb{F}_{2}: [\delta_{1},\delta_{2}] \to \mathbb {R}$ *be a monotonic nondecreasing function*. *Then the inequality*

43 $$ \bigl\vert \mathbb{C}(\mathbb{F}_{1}, \mathbb{F}_{2}) \bigr\vert \le \frac{ \Vert \mathbb{F}_{1}' \Vert _{\infty}}{ 2(\delta _{2}-\delta_{1})} \int_{\delta_{1}}^{\delta_{2}}{(z-\delta_{1}) (\delta _{2}-z)}\,d\mathbb{F}_{2}(z) $$

*holds with the best possible constant* $\frac{1}{2}$.

In the sequel, we consider the above theorems to construct new estimations of generalized identities proved earlier. In what follows, we let for $k=1,2,3,4$,

44 $$ \mathfrak{\tilde{O}}_{k}(v)= \int_{\delta_{1}} ^{\delta_{2}} {\operatorname{POP}\bigl[\mathbf{z}, \mathbf{q};G_{k}(z,w)\bigr]} {{\tilde{R}}_{n - 2}} ( {w,v} )\,dw, \quad v\in[\delta_{1},\delta_{2}] $$

and

45 $$ \mathfrak{O}_{k}(v)= \int_{\delta_{1}} ^{\delta_{2}} {\operatorname {POP}\bigl[\mathbf{z}, \mathbf{q};G_{k}(z,w)\bigr]} {R_{n - 2}} ( {w,v} )\,dw, \quad v \in[\delta_{1},\delta_{2}]. $$

First we express some Ostrowski-type inequalities affiliated with our generalized Popoviciu’s inequality.

### Theorem 10

*Consider the suppositions of Theorem* 4 *be satisfied*. *Let* $\vert \psi^{(n)} \vert ^{r}:[\delta_{1},\delta_{2}]\to \mathbb {R}$ *be an R*-*integrable function for some* $(n\ge4)$ *with* $r,r' \in [1,\infty]$ *such that* $\frac{1}{r}+\frac{1}{r'}=1$. *Then*, *for* $k=1,2,3,4$, *we have*

46 $$\begin{aligned}& \begin{aligned}[b] & \Biggl\vert \operatorname{POP}\bigl[ \mathbf{z},\mathbf{q};\psi(z)\bigr] - \biggl(\frac{{\psi'(\delta_{1} ) - \psi'(\delta_{2} )}}{{\delta_{2} - \delta_{1}} } \biggr) \int_{\delta_{1}}^{\delta_{2}} \operatorname {POP}\bigl[\mathbf{z}, \mathbf{q};G_{k}(z,w)\bigr]\,dw \\ &\qquad{} - \frac{1}{{\delta_{2} - \delta_{1}} } \int_{\delta_{1}} ^{\delta_{2}} \operatorname{POP}\bigl[\mathbf{z}, \mathbf{q};G_{k}(z,w)\bigr]\\ &\qquad{}\times \Biggl({\sum_{l = 2}^{n - 1} {\frac{l}{{(l - 1)!}} \bigl( {{\psi^{ ( l )}} ( \delta_{1} ){{ ( {w - \delta_{1}} )}^{l - 1}} - {\psi^{ ( l )}} ( \delta_{2} ){{ ( {w - \delta_{2}} )}^{l - 1}}} \bigr)}} \Biggr) \,dw \Biggr\vert \\ &\quad\le\frac{1}{ ( n-3 ) !} \bigl\Vert {\psi ^{(n)}} \bigr\Vert _{r} \biggl( \int_{\delta_{1}} ^{\delta_{2}} { \biggl\vert \int_{\delta_{1}} ^{\delta_{2}} {\operatorname{POP}\bigl[\mathbf{z}, \mathbf{q};G_{k}(z,w)\bigr]} {{\tilde{R}}_{n - 2}} ( {w,v} )\,dw \biggr\vert ^{r'}} \,dv \biggr)^{1/r'}, \end{aligned} \end{aligned}$$

47 $$\begin{aligned}& \begin{aligned}[b] & \Biggl\vert {\operatorname{POP}\bigl[ \mathbf{z},\mathbf{q};\psi(z)\bigr]- \biggl( {\frac{{\psi'(\delta_{2} ) - \psi'(\delta_{1} )}}{{\delta_{2} - \delta_{1}} }} \biggr) \int_{\delta_{1}} ^{\delta_{2}} {\operatorname {POP}\bigl[\mathbf{z}, \mathbf{q};G_{k}(z,w)\bigr]} \,dw} \\ &\qquad{} - \frac{1}{{\delta_{2} - \delta_{1}} } \int_{\delta_{1}} ^{\delta_{2}} \operatorname{POP}\bigl[\mathbf{z}, \mathbf{q};G_{k}(z,w)\bigr]\\ &\qquad{}\times \Biggl({\sum_{l = 3}^{n - 1} {\frac{{{\psi^{ ( l )}} (\delta_{1} ){{ ( {w - \delta_{1}} )}^{l - 1}} - {\psi ^{ ( l )}} ( \delta_{2} ){{ ( {w - \delta _{2}} )}^{l - 1}}}}{{(l - 3)!(l - 1)}}}} \Biggr) \,dw\Biggr\vert \\ &\quad\le\frac{1}{ ( n-3 ) !} \bigl\Vert {\psi ^{(n)}} \bigr\Vert _{r} \biggl( \int_{\delta_{1}} ^{\delta_{2}} { \biggl\vert \int_{\delta_{1}} ^{\delta_{2}} {\operatorname{POP}\bigl[\mathbf{z}, \mathbf{q};G_{k}(z,w)\bigr]} {R_{n - 2}} ( {w,v} )\,dw \biggr\vert ^{r'}} \,dv \biggr)^{1/r'}. \end{aligned} \end{aligned}$$

*The constants on the R*.*H*.*S*. *of* (46) *and* (47) *are sharp for* $1< r\le\infty$ *and best possible for* $r=1$.

### Proof

Fix $k=1,2,3,4$. Rearrange identity (16) in such a way that

48 $$ \begin{gathered}[b] \Biggl\vert {\operatorname{POP}\bigl[ \mathbf{z},\mathbf{q};\psi(z)\bigr] - \biggl(\frac{{\psi'(\delta_{1} ) - \psi'(\delta_{2} )}}{{\delta_{2} - \delta _{1}} } \biggr) \int_{\delta_{1}}^{\delta_{2}} {\operatorname{POP}\bigl[\mathbf{z}, \mathbf{q};G_{k}(z,w)\bigr]}\,dw} \\ \qquad{} + \frac{1}{{\delta_{2} - \delta_{1}} } \int_{\delta_{1}} ^{\delta_{2}} \operatorname{POP}\bigl[\mathbf{z}, \mathbf{q};G_{k}(z,w)\bigr]\\ \qquad{}\times \Biggl({\sum_{l = 2}^{n - 1} {\frac{l}{{(l - 1)!}} \bigl( {{\psi^{ ( l )}} ( \delta_{1} ){{ ( {w - \delta_{1}} )}^{l - 1}} - {\psi^{ ( l )}} ( \delta_{2} ){{ ( {w - \delta_{2}} )}^{l - 1}}} \bigr)}} \Biggr) \,dw \Biggr\vert \\ \quad= \frac{1}{(n-3)!} \biggl\vert \int_{\delta_{1}} ^{\delta _{2}}{\tilde{\mathfrak{O}_{k}}( \xi) \psi^{ (n )} (\xi )} \,d\xi \biggr\vert . \end{gathered} $$

Employing the classical Holder’s inequality to R.H.S. of (48) yields (46). The proof for sharpness is similar to Theorem 3.5 in [14] (see also [15]).

The proof of (47) is similar to that of (46), but we utilize identity (17) instead of (16). □

Next we give some upper bounds of Grüss type.

### Theorem 11

*Consider the suppositions of Theorem* 4 *be fulfilled*. *Also let* $\psi^{(n)}$ *be absolutely continuous with* $(\cdot-\delta_{1})(\delta _{2}-\cdot)[\psi^{(n+1)}]^{2}\in L[\delta_{1}, \delta_{2}]$ *such that* $\mathfrak {\tilde{O}}_{k}$, $\mathfrak{O}_{k}$ ($k=1,2,3,4$) *defined in* (44) *and* (45) *respectively*. *Then the remainders* $\operatorname{Rem}(\delta_{1},\delta_{2},\mathfrak {\tilde{O}}_{k},\psi^{(n)})$, $\operatorname{Rem}(\delta_{1},\delta _{2},\mathfrak{O}_{k},\psi^{(n)})$ *given in the following identities*

49 $$\begin{aligned}& \begin{gathered}[b] \operatorname{POP}\bigl[ \mathbf{z},\mathbf{q};\psi(z)\bigr] - \biggl(\frac {{\psi'(\delta_{1} ) - \psi'(\delta_{2} )}}{{\delta_{2} - \delta_{1} }} \biggr) \int_{\delta_{1}}^{\delta_{2}} \operatorname{POP}\bigl[\mathbf{z}, \mathbf{q};G_{k}(z,w)\bigr]\,dw \\ \quad {} - \frac{1}{{\delta_{2} - \delta_{1}} } \int_{\delta_{1}} ^{\delta_{2}}\operatorname{POP}\bigl[\mathbf{z}, \mathbf{q};G_{k}(z,w)\bigr]\\ \quad {}\times \Biggl( {\sum_{l = 2}^{n - 1} {\frac{l}{{(l - 1)!}} \bigl( {{\psi^{ ( l )}} ( \delta_{1} ){{ ( {w - \delta_{1}} )}^{l - 1}} - {\psi^{ ( l )}} ( \delta_{2} ){{ ({w - \delta_{2}} )}^{l - 1}}} \bigr)}} \Biggr) \,dw \\ \quad {}-\frac{\psi^{(n-1)}(\delta_{2})-\psi^{(n-1)}(\delta_{1})}{{(\delta _{2}-\delta_{1}) ( n-3 ) !}} \int_{\delta_{1}} ^{\delta_{2}} {\mathfrak{\tilde{O}}_{k}(v)} \,dv=\operatorname{Rem}\bigl(\delta_{1},\delta _{2}, \mathfrak{\tilde{O}}_{k},\psi^{(n)}\bigr), \end{gathered} \end{aligned}$$

50 $$\begin{aligned}& \begin{gathered}[b] \operatorname{POP}\bigl[ \mathbf{z},\mathbf{q};\psi(z)\bigr]- \biggl( {\frac {{\psi'(\delta_{2} ) - \psi'(\delta_{1} )}}{{\delta_{2} - \delta_{1}} }} \biggr) \int_{\delta_{1}} ^{\delta_{2}} {\operatorname{POP}\bigl[\mathbf{z}, \mathbf{q};G_{k}(z,w)\bigr]} \,dw \\ \quad {} -\frac{1}{{\delta_{2} - \delta_{1}} } \int_{\delta_{1}} ^{\delta_{2}} \operatorname{POP}\bigl[\mathbf{z}, \mathbf{q};G_{k}(z,w)\bigr]\\ \quad {}\times \Biggl( {\sum_{l = 3}^{n - 1} {\frac{{{\psi^{ ( l )}} ( \delta_{1} ){{ ( {w - \delta_{1}} )}^{l - 1}} - {\psi^{ (l )}} ( \delta_{2} ){{ ( {w - \delta_{2}} )}^{l - 1}}}}{{(l - 3)!(l - 1)}}}} \Biggr) \,dw \\ \quad {}-\frac{\psi^{(n-1)}(\delta_{2})-\psi^{(n-1)}(\delta_{1})}{{(\delta _{2}-\delta_{1}) ( n-3 ) !}} \int_{\delta_{1}} ^{\delta_{2}} {\mathfrak{O}_{k}(v)} \,dv= \operatorname{Rem}\bigl(\delta_{1},\delta _{2}, \mathfrak{O}_{k},\psi^{(n)}\bigr), \end{gathered} \end{aligned}$$

*satisfy the bounds*

$$ \begin{aligned} &\bigl\vert \operatorname{Rem}\bigl(\delta_{1},\delta_{2}, \mathfrak{\tilde {O}}_{k},\psi^{(n)}\bigr) \bigr\vert \\ &\quad \le \frac{1}{(n-3)!} \bigl[\mathbb {C}(\mathfrak{\tilde{O}}_{k},\mathfrak{ \tilde{O}}_{k}) \bigr]^{\frac {1}{2}}\sqrt{\frac{(\delta_{2}-\delta_{1})}{2}} \biggl\vert \int _{\delta_{1}}^{\delta_{2}}{(v-\delta_{1}) ( \delta_{2}-v)\bigl[\psi ^{(n+1)}(v)\bigr]^{2}}\,dv \biggr\vert ^{\frac{1}{2}} \end{aligned} $$

*and*

$$ \begin{aligned} &\bigl\vert \operatorname{Rem}\bigl(\delta_{1},\delta_{2}, \mathfrak {O}_{k},\psi^{(n)}\bigr) \bigr\vert \\ &\quad \le \frac{1}{(n-3)!} \bigl[\mathbb {C}(\mathfrak{O}_{k}, \mathfrak{O}_{k}) \bigr]^{\frac{1}{2}}\sqrt{\frac {(\delta_{2}-\delta_{1})}{2}} \biggl\vert \int_{\delta_{1}}^{\delta _{2}}{(v-\delta_{1}) ( \delta_{2}-v)\bigl[\psi^{(n+1)}(v)\bigr]^{2}}\,dv \biggr\vert ^{\frac{1}{2}}, \end{aligned} $$

*respectively*.

### Proof

Fix $k=1,2,3,4$. Using Čebyšev’s functional for $\mathbb {F}_{1}=\mathfrak{O}_{k}$, $\mathbb{F}_{2}=\psi^{(n)}$ and by comparing (49) with (16), we have

$$\operatorname{Rem}\bigl(\delta_{1},\delta_{2},\mathfrak{ \tilde{O}}_{k},\psi ^{(n)}\bigr)=\frac{\delta_{2}-\delta_{1}}{(n-3)!}\mathbb{C} \bigl(\mathfrak {\tilde{O}}_{k},\psi^{(n)}\bigr). $$

Now applying Theorem 8 for the corresponding functions, we get the required bound.

Similarly, by comparing (50) with identity (17), we get the respective bound. □

### Theorem 12

*Consider the suppositions of Theorem* 4 *be fulfilled*, *and let* $\psi^{(n+1)}\ge0$ *on* $[\delta_{1},\delta_{2}]$ *with* $\mathfrak{\tilde {O}}_{k}$, $\mathfrak{O}_{k}$ ($k=1,2,3,4$) *defined in* (44) *and* (45), *respectively*. *Then in representation* (49) *the remainder* $\operatorname {Rem}(\delta_{1},\delta_{2},\mathfrak{\tilde{O}}_{k},\psi^{(n)})$ *satisfies the estimation*

51 $$ \begin{gathered}[b] \bigl\vert \operatorname{Rem}\bigl(\delta_{1}, \delta_{2},\mathfrak{\tilde {O}}_{k},\psi^{(n)}\bigr) \bigr\vert \\ \quad \le\frac{ (\delta_{2}-\delta_{1}) \Vert \mathfrak{\tilde{O}}_{k}' \Vert _{\infty}}{ (n-3 ) !} \biggl[\frac{\psi^{(n-1)}(\delta_{2})+\psi ^{(n-1)}(\delta_{1})}{2}-\frac{\psi^{(n-2)}(\delta_{2})-\psi ^{(n-2)}(\delta_{1})}{\delta_{2}-\delta_{1}} \biggr], \end{gathered} $$

*whereas in representation* (50) *the remainder* $\operatorname {Rem}(\delta_{1},\delta_{2},\mathfrak{O}_{k},\psi^{(n)})$ *satisfies the estimation*

52 $$ \begin{gathered}[b] \bigl\vert \operatorname{Rem}\bigl(\delta_{1}, \delta_{2},\mathfrak {O}_{k},\psi^{(n)}\bigr) \bigr\vert \\ \quad \le\frac{ (\delta_{2}-\delta_{1}) \Vert \mathfrak{O}_{k}' \Vert _{\infty}}{ ( n-3 ) !} \biggl[\frac{\psi^{(n-1)}(\delta_{2})+\psi^{(n-1)}(\delta _{1})}{2}-\frac{\psi^{(n-2)}(\delta_{2})-\psi^{(n-2)}(\delta _{1})}{\delta_{2}-\delta_{1}} \biggr]. \end{gathered} $$

### Proof

Fix $k=1,2,3,4$. We have established

$$\operatorname{Rem}\bigl(\delta_{1},\delta_{2},\mathfrak{ \tilde{O}}_{k},\psi ^{(n)}\bigr)=\frac{1}{(n-3)!}\mathbb{C} \bigl(\mathfrak{\tilde{O}}_{k},\psi^{(n)}\bigr). $$

Now applying Theorem 9 for $\mathbb{F}_{1}\rightarrow \mathfrak{O}_{k}$ and $\mathbb{F}_{2}\rightarrow\psi^{(n)}$, we have

53 $$\begin{aligned} \bigl\vert \operatorname{Rem}\bigl(\delta_{1}, \delta_{2},\mathfrak {O}_{k},\psi^{(n)}\bigr) \bigr\vert =&\frac{1}{(n-3)!} \bigl\vert \mathbb {C}\bigl(\mathfrak{ \tilde{O}}_{k},\psi^{(n)}\bigr) \bigr\vert \\ \le&\frac{ \Vert \mathfrak{O}_{k}' \Vert _{\infty}}{2(\delta_{2}-\delta_{1})(n-3)!} \int_{\delta_{1}}^{\delta_{2}}{(\xi-\delta _{1}) ( \delta_{2}-\xi)}\psi^{(n+1)}(\xi). \end{aligned}$$

Simplifying the integral on R.H.S. of (53), we get the estimation in (52). □

## Mean value theorems and *n*-exponential convexity

In the present section, we construct positive linear functionals and then give mean value theorems of Lagrange and Cauchy type.

### Remark 7

By virtue of Theorem 5, we can define the positive linear functionals from (22) ($k=1,2,3,4$), with respect to *n*-convex function *ψ* as follows:

54 $$\begin{aligned}& \begin{aligned}[b] \Omega_{k}(\psi)&:= \operatorname{POP}\bigl[\mathbf{z},\mathbf{q};\psi(z)\bigr]\\ & \ge \biggl( \frac{{\psi'(\delta_{1} ) - \psi'(\delta_{2} )}}{{\delta_{2} - \delta_{1}} } \biggr) \int_{\delta_{1}}^{\delta_{2}} {\operatorname {POP}\bigl[\mathbf{z}, \mathbf{q};G_{k}(z,w)\bigr]}\,dw \\ &\quad{} - \frac{1}{{\delta_{2} - \delta_{1}} } \int_{\delta_{1}} ^{\delta_{2}} \operatorname{POP}\bigl[\mathbf{z}, \mathbf{q};G_{k}(z,w)\bigr]\\ &\quad {}\times \Biggl({\sum_{l = 2}^{n - 1} {\frac{l}{{(l - 1)!}} \bigl( {{\psi^{ ( l )}} ( \delta_{1} ){{ ( {w - \delta_{1}} )}^{l - 1}} - {\psi^{ ( l )}} ( \delta_{2} ){{ ( {w - \delta_{2}} )}^{l - 1}}} \bigr)}} \Biggr) \,dw . \end{aligned} \end{aligned}$$

55 $$\begin{aligned}& \begin{aligned}[b] \Delta_{k}(\psi)&:= \operatorname{POP}\bigl[\mathbf{z},\mathbf{q};\psi(z)\bigr] - \biggl( { \frac{{\psi'(\delta_{2} ) - \psi'(\delta_{1} )}}{{\delta_{2} - \delta_{1}} }} \biggr) \int_{\delta_{1}} ^{\delta_{2}} {\operatorname {POP}\bigl[\mathbf{z}, \mathbf{q};G_{k}(z,w)\bigr]} \,dw \\ &\quad{} - \frac{1}{{\delta_{2} - \delta_{1}} } \int_{\delta_{1}} ^{\delta_{2}} \operatorname{POP}\bigl[\mathbf{z}, \mathbf{q};G_{k}(z,w)\bigr]\\ &\quad{}\times \Biggl(\sum_{l = 3}^{n - 1} \frac{{{\psi^{ ( l )}} (\delta_{1} ){{ ( {w - \delta_{1}} )}^{l - 1}} - {\psi ^{ ( l )}} ( \delta_{2} ){{ ( {w - \delta _{2}} )}^{l - 1}}}}{{(l - 3)!(l - 1)}} \Biggr) \,dw. \end{aligned} \end{aligned}$$

We give results related to $\Omega_{k}$ as we can also consider $\Delta _{k}$ in the similar fashion for ($k=1,2,3,4$). Lagrange- and Cauchy-type mean value theorems related to the above functionals are given in the following theorems.

### Theorem 13

*Let* $\psi:[\delta_{1},\delta_{2}]\rightarrow \mathbb {R}$ *be such that* $\psi\in C^{n}[\delta_{1},\delta_{2}]$. *If the inequality in* (21) ($k=1,2,3,4$) *holds*, *then there exist* $\xi_{k}\in [\delta_{1},\delta_{2}]$ *such that*

$$ \Omega_{k}(\psi)=\psi^{(n)}(\xi_{k}) \Omega_{k}\biggl(\frac{z^{n}}{n!}\biggr), \quad k=1,2,3,4, $$

*where* $\Omega_{k}(\cdot)$ *is defined by* (54).

### Proof

Similar to the proof of Theorem 4.1 in [16] (see also [17]). □

### Theorem 14

*Let* $\psi,\mu:[\delta_{1},\delta_{2}]\rightarrow \mathbb {R}$ *be such that* $\psi,\mu\in C^{n}[\delta_{1},\delta_{2}]$. *If the inequality in* (21) ($k=1,2,3,4$) *holds*, *then there exist* $\xi_{k}\in[\delta_{1},\delta_{2}]$ *such that*

$$ \frac{\Omega_{k}(\psi)}{ \Omega_{k}(\mu)}=\frac{\psi^{(n)}(\xi_{k})}{\mu^{(n)}(\xi_{k})}, \quad k=1,2,3,4, $$

*provided that the denominators are non*-*zero*, *where* $\Omega_{k}(\cdot)$ *is defined by* (54).

### Proof

Similar to the proof of Corollary 4.2 in [16] (see also [17]). □

Theorem 14 enables us to define Cauchy means for ($k=1,2,3,4$), in fact

$${\xi_{k}} = { \biggl( {\frac{{{\psi^{(n)}}}}{{{\mu^{(n)}}}}} \biggr)^{ - 1}} \biggl( {\frac{{{\Omega_{k}} (\psi)}}{{{\Omega_{k}} (\mu)}}} \biggr), $$

means that *ξ* is the mean of $\delta_{1}$, $\delta_{2}$ for given functions *ψ* and *μ*.

We conclude our paper with the following remark.

### Remark 8

One can also construct the nontrivial examples of *n*-exponentially and exponentially convex functions from positive linear functionals $\Omega_{k}(\cdot)$ and $\Delta_{k}(\cdot)$ ($k=1,2,3,4$) by following the *n*-exponential method introduced by Pečarić et al. in [18] and [19] (see also [14, 20] and [15]). As an application it enables us to construct large families of functions which are exponentially convex. Moreover, by considering the class of 2-convex functions, we can get the log-convexity of these functionals and new Cauchy means, which are monotonic in nature.

## Conclusions

By integration techniques new Green’s functions are constructed, which are convex symmetric and continuous. Graphical representation of these new Green’s functions is also included. These new Green’s functions are then used to extend the inequality of Popoviciu given by Vasić and Stanković from nonnegative to real weights. Generalized identities are obtained using generalized Montgomery identity and new Green’s functions which further establish the extension of Popoviciu inequality from a convex function to higher order convex functions along with real weights. The obtained results are then applied to establish the monotonicity of the linear functionals constructed from generalized inequalities. New upper bounds are obtained using the Čebyšev functional. A new way is introduced to construct new *n*-exponential and logarithmic convex functions, which are then further used to give the Stolarsky means.

## References

[1] Vasić PM, Stanković LR (1976). Some inequalities for convex functions. Math. Balk..

[2] Pečarić J, Proschan F, Tong YL (1992). Convex Functions, Partial Orderings and Statistical Applications.

[3] Butt SI, Pecaric J (2016). Popoviciu’s Inequality for n-Convex Functions.

[4] Pečarić J, Praljak M, Witkowski A (2015). Linear operator inequality for *n*-convex functions at a point. Math. Inequal. Appl..

[5] Popoviciu T (1965). Sur certaines inégalités qui caractérisent les fonctions convexes. An. ştiinţ. Univ. ‘Al.I. Cuza’ Iaşi, Mat..

[6] Bencze M, Niculescu CP, Popovici F (2010). Popoviciu’s inequality for functions of several variables. J. Math. Anal. Appl..

[7] Mihai MV, Mitroi-Symeonidis FC (2016). New extensions of Popoviciu’s inequality. Mediterr. J. Math..

[8] Niculescu CP (2009). The integral version of Popoviciu’s inequality. J. Math. Inequal..

[9] Niculescu CP, Popovici F (2006). A refinement of Popoviciu’s inequality. Bull. Math. Soc. Sci. Math. Roum..

[10] Aglić Aljinović A, Pečarić J, Vukelić A (2005). On some Ostrowski type inequalities via Montgomery identity and Taylor’s formula II. Tamkang J. Math..

[11] Mitrinović DS, Pečarić JE, Fink AM (1994). Inequalities for Functions and Their Integrals and Derivatives.

[12] Agarwal RP, Wong PJY (1993). Error Inequalities in Polynomial Interpolation and Their Applications.

[13] Cerone P, Dragomir SS (2014). Some new Ostrowski-type bounds for the Čebyšev functional and applications. J. Math. Inequal..

[14] Butt SI, Khan KA, Pečarić J (2016). Further generalization of Popoviciu inequality for higher order convex functions via Taylor polynomial. Turk. J. Math..

[15] Butt SI, Khan KA, Pečarić J (2015). Popoviciu type inequalities via Green function and generalized Montgomery identity. Math. Inequal. Appl..

[16] Jakšetić J, Pečarić J, Perušić A (2014). Steffensen inequality, higher order convexity and exponential convexity. Rend. Circ. Mat. Palermo.

[17] Butt SI, Pečarić J (2013). Generalized Hermite-Hadamard’s inequality. Proc. A. Razmadze Math. Inst..

[18] Jakšetić J, Pečarić J (2013). Exponential convexity method. J. Convex Anal..

[19] Pečarić J, Perić J (2012). Improvement of the Giaccardi and the Petrović inequality and related Stolarsky type means. An. Univ. Craiova, Ser. Mat. Inform..

[20] Butt SI, Pečarić J, Vukelić A (2016). Generalization of Popoviciu type inequalities via Fink’s identity. Mediterr. J. Math..

